# Diagnostic Accuracy of Ultrasound Scanning for Prenatal Microcephaly in the context of Zika Virus Infection: A Systematic Review and Meta-analysis

**DOI:** 10.1038/s41598-017-01991-y

**Published:** 2017-05-23

**Authors:** Ezinne C. Chibueze, Alex J. Q. Parsons, Katharina da Silva Lopes, Takemoto Yo, Toshiyuki Swa, Chie Nagata, Nobuyuki Horita, Naho Morisaki, Olukunmi O. Balogun, Amarjargal Dagvadorj, Erika Ota, Rintaro Mori, Olufemi T. Oladapo

**Affiliations:** 1 Department of Health Policy, National Center for Child Health and Development, Tokyo, Japan; 2 Department of Global Health Policy, Graduate School of Medicine, University of Tokyo, Tokyo, Japan; 3 Graduate School of Human Sciences, Osaka University, Osaka, Japan; 4 Department of Education for Clinical Research, National Center for Child Health and Development, Tokyo, Japan; 5 Pulmonology, Graduate School of Medicine, Yokohama City University, Yokohama, Japan; 6 Department of Social Medicine, National Center for Child Health and Development, Tokyo, Japan; 7 Department of Health Informatics, Kyoto University, Yoshida Konoe-cho, Syako-ku, Kyoto, Japan; 8 St. Luke’s International University, Graduate school of Nursing, Global Health Nursing, Tokyo, Japan; 9 UNDP/UNFPA/UNICEF/WHO/World Bank Special Programme of Research, Development and Research Training in Human Reproduction (HRP), Department of Reproductive Health and Research World Health Organization, Geneva, Switzerland

## Abstract

To assess the accuracy of ultrasound measurements of fetal biometric parameters for prenatal diagnosis of microcephaly in the context of Zika virus (ZIKV) infection, we searched bibliographic databases for studies published until March 3rd, 2016. We extracted the numbers of true positives, false positives, true negatives, and false negatives and performed a meta-analysis to estimate group sensitivity and specificity. Predictive values for ZIKV-infected pregnancies were extrapolated from those obtained for pregnancies unrelated to ZIKV. Of 111 eligible full texts, nine studies met our inclusion criteria. Pooled estimates from two studies showed that at 3, 4 and 5 standard deviations (SDs) <mean, sensitivities were 84%, 68% and 58% for head circumference (HC); 76%, 58% and 58% for occipitofrontal diameter (OFD); and 94%, 85% and 59% for biparietal diameter (BPD). Specificities at 3, 4 and 5 SDs below the mean were 70%, 91% and 97% for HC; 84%, 97% and 97% for OFD; and 16%, 46% and 80% for BPD. No study including ZIKV-infected pregnant women was identified. OFD and HC were more consistent in specificity and sensitivity at lower thresholds compared to higher thresholds. Therefore, prenatal ultrasound appears more accurate in detecting the absence of microcephaly than its presence.

## Introduction

Microcephaly is a sign of fetal brain abnormality in which there is a significantly small head size for gestational age and sex. Infants born with microcephaly are likely to present with variable clinical features ranging from subtle impairment in neurological development to serious intellectual disabilities in the long term. It is a rare condition occurring in 5.8 to 18.7 per 100,000 pregnancies and often arising from a wide variety of conditions that can cause abnormal brain growth^1^.

In 2015, a 20-fold increase in neonatal microcephaly was observed in association with Zika virus (ZIKV) infections in pregnant women in Latin America^2^. This observation prompted the World Health Organization (WHO) to declare the ZIKV outbreak in the Americas a *Public Health Emergency of International Concern* on 1st February 2016^3^.

As part of its strategic framework, WHO provides normative guidance to affected countries on conditions presumably associated with prenatal ZIKV infection, to improve surveillance and clinical outcomes in at risk populations. The WHO interim guidance recommends that pregnant women residing in areas of ongoing ZIKV transmission should have fetal ultrasound scans to exclude microcephaly or other brain abnormalities that have been reported in fetuses of women with prenatal ZIKV infection^4^.

Prenatal assessment of microcephaly has conventionally relied on ultrasound measurements of fetal biometric parameters such as the head circumference, biparietal diameter and occipitofrontal diameter^5–7^. The measurements of these parameters below a given threshold and at a specific gestational age of assessment have been applied to diagnose fetal microcephaly^6, 8^. However, at the time of this review, no international consensus on fetal biometric parameters or the threshold for in-utero microcephaly diagnosis exists. Also, due to the rare nature of this condition, the application of different parameters and limits, the risk of wrong or missed diagnosis is high^9–12^.

In the context of ZIKV infection, an accurate prenatal diagnosis of microcephaly is critical for fetal prognosis and decision-making by health providers and families of women suspected or confirmed to have ZIKV infection. We conducted a systematic review to assess the diagnostic accuracy of ultrasound measurement of fetal biometric parameters compared to reference assessments at birth for prenatal diagnosis of microcephaly in the context of ZIKV infection. This review served as part of the evidence base for the revised WHO interim guidance on the prenatal assessment of microcephaly in the context of ZIKV infection.

## Methodology

### Protocol registration

We registered this review in PROSPERO, the international prospective register of systematic reviews of the University of York and the National Institute for Health Research, under the number CRD42016039365.

### Search strategies

We searched MEDLINE, EMBASE, CENTRAL, Cochrane Database for DTA studies, LILACS, and WHO Global Health Library for studies published until 3rd March 2016. Search terms related to the index tests, reference tests and target condition were employed in the search strategies as shown in Appendix 1 (Supplementary Information).

Searches for grey literature and bibliographies of existing systematic reviews on ultrasound in pregnancy were complemented with results of the search strategies. No restrictions were placed on search dates or language. Two review authors independently screened the titles and abstracts of studies identified by the search strategies. Full texts of potentially eligible studies were independently assessed by two review authors for relevant studies.

Any disagreements were resolved through discussion or consultation with a third review author.

### Inclusion and exclusion criteria

#### Index tests, reference standard, and diagnosis of interest

We considered studies that compared prenatal ultrasound measurements (index test) with direct postnatal measurements of head size (reference test). We included studies which used any of the following biometric parameters as index tests: head circumference (HC), occipitofrontal diameter (OFD), biparietal diameter (BPD), or ratios of any of these with either abdominal circumference (AC) or femur length (FL).

Microcephaly (diagnosis) was the condition of interest, reported either as the only condition or separately in addition to other fetal brain abnormalities.

#### Types of studies

We considered for inclusion, studies of any design (randomized controlled trial, prospective or retrospective cohort studies, cross-sectional studies and case-control studies) comparing prenatal assessment of fetal biometric parameters with standard postnatal head size measurements for diagnosing microcephaly.

Case series and conference proceedings reporting original data and with adequate information were also considered for inclusion.

#### Types of participants

Pregnant women who had ultrasound measurements of fetal biometric parameters for diagnosis of microcephaly (irrespective of the indications for ultrasound). We planned to separately assess pregnant women suspected of being at risk of or confirmed with ZIKV infection.

### Data extraction and synthesis

Two review authors independently extracted data on participants’ characteristics (ZIKV virus infection status, gestational age at the time of ultrasound assessment). We obtained data on the number of true positives (TP), false positives (FP), true negatives (TN) and false negatives (FN) to determine the sensitivity and specificity (with 95% confidence intervals [CI]) of the index tests for each fetal biometric parameter.

Results from studies that presented insufficient data for meta-analysis were qualitatively shown. In one study where ultrasound and magnetic resonance imaging (MRI) were employed^13^, only sonographic data was extracted for the review.

For studies considered similar in terms of the research questions, study design and execution, we performed a meta-analysis using a random effects model on pooled data to estimate group sensitivity and specificity (with 95% CI).

We generated hierarchical receiver operating characteristic (HROC) curves using a hierarchical summary receiver operating characteristic (HSROC) model. To gauge overall test accuracy, we calculated a diagnostic odds ratio (DOR) and an area under the curve (AUC) using Der Simonian-Laird random-modeling and Holling’s proportional hazard model^14^.

Data on TP, TN, FP and FN using cut-off values ranging from 3 SD to 5 SD below the mean were applied to estimate diagnostic test accuracy of fetal ultrasound. Pre-test probabilities based on the incidence of microcephaly in unclassified pregnancy (0.0285%)^1^ and ZIKV-infected pregnancies (0.95%)^1^ were applied to estimate positive and negative predictive values.

### Risk of bias assessment

We assessed the risk of bias using version 2 of the Quality Assessment of Diagnostic Accuracy Studies (QUADAS-2) tool^15, 16^ in Review Manager (RevMan Version 5.3.). We provided a rating for risk of bias and applicability concerns based on the presence or absence of indicators for index and reference standards, flow and timing of prenatal and postnatal tests.

We assessed as high risk where serious deficiencies in criteria were detected and unclear or low risk where descriptions were inadequate or appropriate. For the meta-analysed data, we assessed heterogeneity using the I^2^ statistic (percentage of inter-study variation due to heterogeneity).

## Results

### Search results

**Figure 1:**
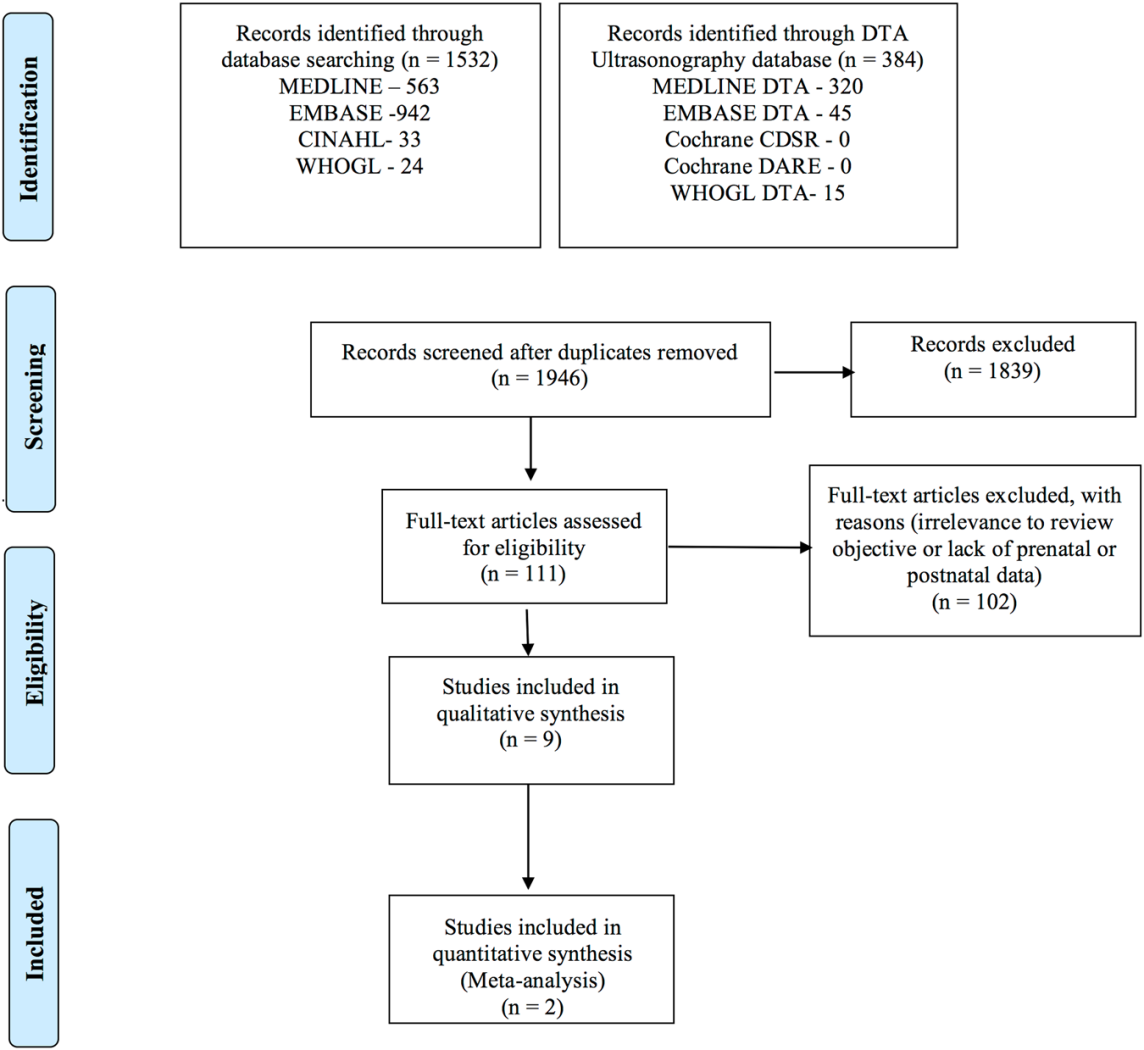
Flow diagram. Search results and study selection (see appendices for details).

The search strategies yielded 2,258 citations from the databases. One hundred and eleven potentially eligible studies were identified after screening of titles and abstracts and removing duplicates (Fig. 1).

Full texts of all potentially eligible studies were assessed and studies excluded for reasons shown (Appendix 2, Supplementary Information). Nine studies met our inclusion criteria. Two of these studies reported sufficient data that could be used in meta-analysis while the other seven presented incomplete data and were described.

### Characteristics of included studies

All included studies were based on hospital records in the USA (5), Israel (2), France (1) and Canada (1). The study designs were either prospective cohort^17–20^ or retrospective cohort^13, 21–24^ with enrollment periods spanning between 1979 and 2014 (Table 1).

**Table 1:** Characteristics of included studies.

Author and year	Country	Enrollment period	Setting (e.g. facility, medical records)	Study design	Participant information	Index test	Reference test	Reported outcomes	Ultrasound device
Campbell^23^	USA	1978 to June 1983	Hospital records	Retrospective	10 cases correctly detected on the basis before 26 weeks gestation, with no false positives and no false negatives based on two parameters with prenatal and postnatal confirmation implied	AC HC	AC HC	Microcephaly (unclear definition)	Not provided
Chervanak^18^	USA	July 1, 1979, to July 1, 1983	Medical Center, medical records	Prospective	16 fetuses (initially 18, two were later excluded as they were stillbirths)	BPD OFD HC HC: AP BPD: FL FL: HC	BPD OFD HC	Microcephaly (defined as a HC of <3 SDs below the mean for gestational age at birth)	Not reported
Chervenak^19^	USA	1983–1986	Medical Center	Prospective	Prenatal diagnosis was done for 24 fetuses using different biometrical parameters	BPD OFD HC HC: AP FL: HC	BPD OFD HC HC: AP FL: HC	Microcephaly (defined as an occipitofrontal diameter (OFD) of smaller than the predicted mean -3SD at birth) Deaths Stillbirths, Encephalocele	Not reported
Wilson^24^	Canada	1982 to 1985	Hospital, medical records	Retrospective	16 cases identified prenatally were assessed for abnormalities	HC	HC (postnatal assessment)	Microcephaly (defined as a HC of 3 SD below normal at birth)	Not provided
Harvey L^20^	USA	Unknown	Maternal PKU Collaborative Study (MPKUCS) database	Prospective	31 fetuses in the second trimester and 20 in the third trimester, all from pregnant mothers diagnosed with phenylketonuria (PKU) and limited to live births	BPD	BPD	Microcephaly (defined as a fetal BPD of >3 SD below the mean)	Acuson 128 XP 10 (Mountain View, CA, U.S.A.) scanner with a 3–5 or 5 MHz variable focus transducer.
Benoist^13^	France	2000–2007	Hospital, medical records	Retrospective	49 fetuses of CMV-infected mothers, prenatal ultrasound investigations were compared to postnatal investigations (both autopsy and live births). 38 live births, ten terminations of pregnancy and one fetal death	HC; Serial targeted transabdominal or transvaginal ultrasound of the HC (every fortnight from diagnosis until delivery)	HC: Transfontanellar ultrasound measurement of HC at birth or postmortem findings on fetal autopsy	Microcephaly (defined as a fetal HC of <5th percentile for gestational age)	GE Voluson 730 ultrasound examinations with high-frequency probes (transabdominal for breech presentation (4–8 MHz) and transvaginal for normal presentation (5–9 MHz) applications) GE Medical Systems, Ultrasound and Primary Care Diagnostic, Gif Sur Yvette, France)
Stoler-Poria^17^	Israel	2001 to 2005	Medical Center	Prospective	20 fetuses were included and followed up for neurodevelopment outcomes	HC	Postnatal HC	Microcephaly (fetal head circumference measure >2 SD below the gestational mean), developmental outcome, neurological development, microcephaly	Not provided
Wong^22^	USA	January 2005 to July 2011	Hospital, medical charts	Retrospective	730 ultrasounds of 455 fetuses in 433 patients	HC	Birth HC	Microcephaly (defined as a HC of <10 percentile at birth)	Not provided
Leibovitz^21^	Israel	2007 to 2014	Hospital, medical records	Retrospective	42 fetuses were evaluated	BPD OFD HC (1.62 (BPD + OFD)) HC:AP FL:HC	HC	Microcephaly (defined as a fetal HC of <3 SDs below the mean for gestational age; Chervenak *et al*.^18^ was used as a reference) Normocephaly	Voluson E8, Voluson 730 Expert, and Voluson 730 Pro ultrasound machines (GE Healthcare Ultrasound, Milwaukee, WI, USA)

**Abbreviations:** Abdominal circumference (AC), Biparietal Diameter (BPD), Femur Length (FL), Head Circumference (HC), Microcephaly (MCP), Ultrasound (US), Occipitofrontal Diameter (OFD), Standard Deviation (SD).

The thresholds for prenatal and postnatal diagnoses of microcephaly were pre-specified in some studies. It was defined as head measurements of >2 SD below the mean^17^, <3 SD below the mean^18, 19, 21^, 3 SD below the mean^24^, >3 SD below the mean^20^, below the 5th percentile^13^ and the 10th percentile^22^ threshold. The threshold applied was unstated in one study^23^.

**Figure 2:**
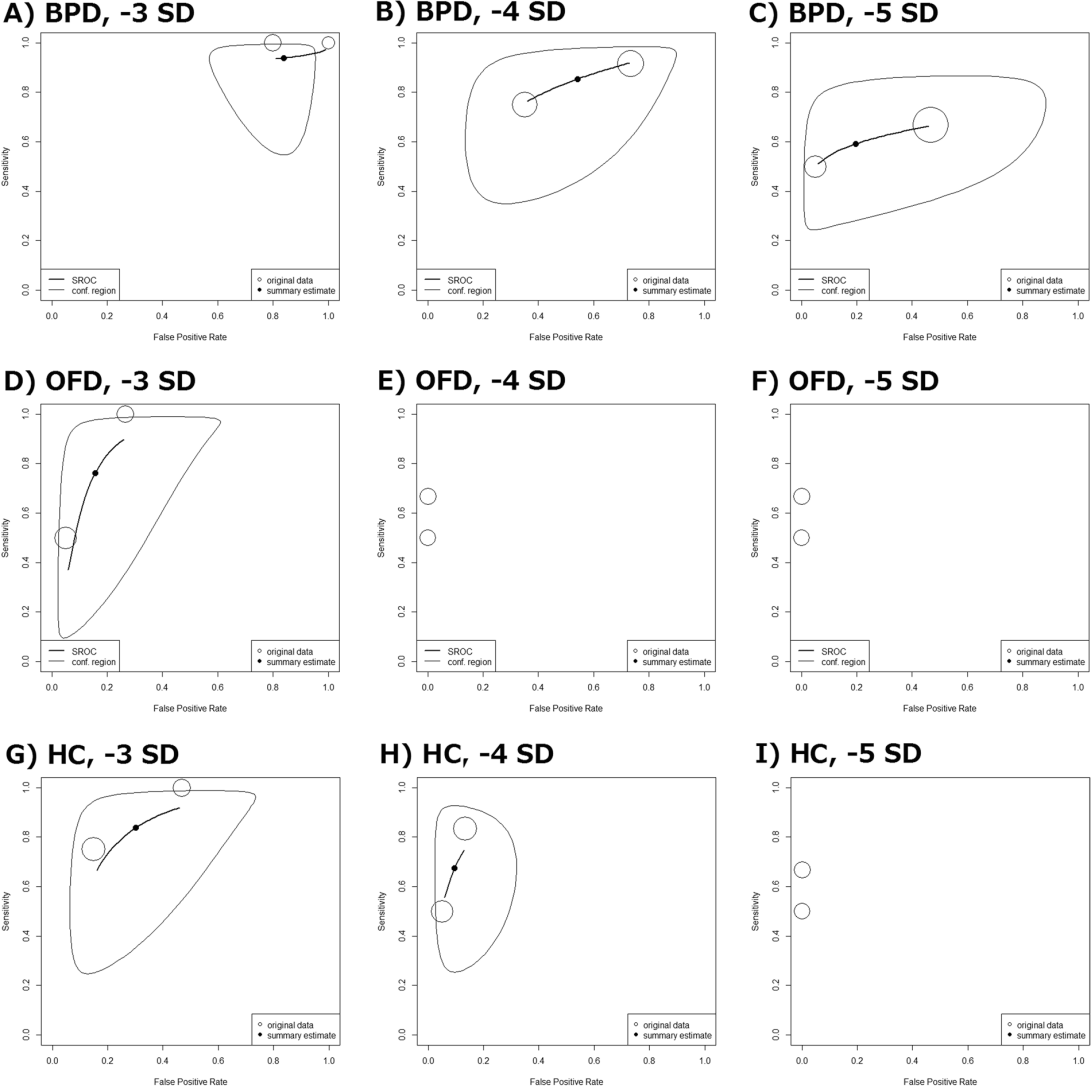
Hierarchical summary receiver operating characteristics (HSROC) curves for **A–C**) BPD, **D–F**) OFD and **G–I**) HC at 3, 4 and 5 SD below the mean. The size of each circle reflects weight, not confidence region. (Open arrow: Two circles had exactly same accuracy and weight. Filled arrow: Three circles had exactly same accuracy and weight).

Only two studies contained data appropriate for meta-analysis as they assessed similar parameters at same thresholds^18, 19^. The HSROC curves are shown in Fig. 2A–I.

Fetal microcephaly was secondary to cytomegalovirus (CMV)-infection^13^ and phenylketonuria (PKU)^20^ in 2 studies and congenital or primary in the seven other studies^17–19, 21–24^.

In three out of nine studies^13, 20, 21^, the ultrasound device used for prenatal detection of fetal parameters was reported. These included Acuson 128 XP 10 (Siemens)^20^, GE Voluson 730^13^ and a range of ultrasound machines in the third study^21^: GE Voluson E8, 730 Expert and Voluson 730 Pro (all GE Healthcare).

### Accuracy of ultrasound measurements of BPD (3 studies)

Meta-analysis of two studies^18, 19^, which included 51 fetuses reported a high sensitivity (94%) at 3 SD below the mean but lower sensitivities at 4 and 5 SDs. The specificity at 3 SD was very low but improved with lower cut-offs. The positive likelihood ratio for 3 SD suggests a slight increase in the likelihood of microcephaly, but the confidence interval includes 1 (suggesting no change in the likelihood of microcephaly) (Table 2, Fig. 2A–C).

**Table 2:** Diagnostic accuracy of ultrasound measurements of BPD for prenatal assessment of microcephaly.

Cut-off	−3 SD	−4 SD	−5 SD
Number of cohorts	2^18, 19^	2^18, 19^	2^18, 19^
Number of comparisons	51	51	51
Diagnostic odds ratio (DOR)	1.6 (0.056–46.1), I^2^ = 0%	4.7 (0.86–25.5), I^2^ = 0%	4.7 (0.66–33.9), I^2^ = 0%
AUC	0.888	0.77	0.66
Sensitivity	0.94 (0.67–0.99)	0.85 (0.46–0.98)	0.59 (0.30–0.83)
Specificity	0.16 (0.06–0.37)	0.46 (0.14–0.81)	0.80 (0.21–0.99)
Positive likelihood ratio	1.1 (0.82–1.5)	1.6 (0.70–4.5)	3.0 (0.59–46.0)
Negative likelihood ratio	0.38 (0.047–2.5)	0.33 (0.045–1.9)	0.51 (0.21–2.6)
PPV (general pregnancy)	0.00032	0.00045	0.00084
NPV (general pregnancy)	0.99989	0.99991	0.99985
PPV (ZIKV-infected pregnancy)	0.0106	0.0109	0.0275
NPV (ZIKV-infected pregnancy)	0.9964	0.9969	0.995

Parentheses indicate 95% CI. Pre-test probabilities, i.e. incidence of microcephaly among general pregnancies and ZIKV-infected pregnancies were estimated as 0.0285% and 0.95%, respectively.

The positive likelihood ratios for 4 and 5 SDs indicate a large and often conclusive increase in the likelihood of microcephaly with the ratios exceeding 1. The positive predictive values (PPV) for unspecified and ZIKV-infected pregnancies were even much lower than for OFD measurements across the three thresholds.

One study^20^ provided descriptive data. This study noted a low true positive and a high false negative frequency for the second (3.2%; 29%) and third trimester (42.9%; 57.1%) at a threshold of 3 SD below the mean.

### Accuracy of ultrasound measurement of OFD (2 studies)

Pooled data from two studies^18, 19^ (45 fetuses) reported sensitivities of 76%, 58% and 58% and specificity of 84%, 97% and 97% at 3, 4, and 5 SDs below the mean for GA, respectively. Higher thresholds were more sensitive while lower thresholds were more specific (Table 3, Fig. 2D–F).

**Table 3:** Diagnostic accuracy of ultrasound measurements of OFD for prenatal assessment of microcephaly.

Cut-off	−3 SD	−4 SD	−5 SD
Number of cohorts	2^18, 19^	2^18, 19^	2^18, 19^
Number of comparisons	45	45	45
Diagnostic odds ratio	18.6 (2.8–124.2), I^2^ = 0%	48.0 (4.8–481.5), I^2^ = 0%	48.0 (4.8–481.5), I^2^ = 0%
AUC	0.88	0.68	0.68
Sensitivity	0.76 (0.17–0.98)	0.58 (0.30–0.82)	0.58 (0.30–0.82)
Specificity	0.84 (0.50–0.97)	0.97 (0.83–1.00)	0.97 (0.83–1.00)
Positive likelihood ratio	4.8 (0.73–23.3)	19.3 (3.0–126.3)	19.3 (3.0–126.3)
Negative likelihood ratio	0.29 (0.024–1.1)	0.43 (0.19–0.74)	0.43 (0.19–0.74)
PPV (general pregnancy)	0.00135	0.00548	0.00548
NPV (general pregnancy)	0.99992	0.99988	0.99988
PPV (ZIKV-infected pregnancy)	0.0436	0.1564	0.1564
NPV (ZIKV-infected pregnancy)	0.9973	0. 9959	0.9959

Parentheses indicate 95% CI. Pre-test probabilities, i.e. incidence of microcephaly among general pregnancies and ZIKV-infected pregnancies were estimated as 0.0285% and 0.95%, respectively.

OFD measurement at a threshold of 3 SD below the mean for GA was more sensitive, and measurements at 4 and 5 SDs more specific. Given the extremely low incidence of microcephaly applied, the proportion of fetuses diagnosed with microcephaly based on 3, 4, and 5 SD thresholds which were correctly diagnosed (PPV) was extremely low. Deduction of PPVs using 0.95% incidence of microcephaly among ZIKV-infected women did improve the PPV (Table 3, Fig. 2D–F).

However, the proportion of fetuses without microcephaly who were correctly diagnosed was close to 100% for the three thresholds, for both unspecified and ZIKV-infected pregnancies.

### Accuracy of ultrasound measurements of HC (8 studies)

Eight studies reported on the diagnostic accuracies of HC. Synthesis of two studies^18, 19^ (45 fetuses) with meta-analyzable data showed sensitivities of 84%, 68% and 58% and specificity of 70%, 91% and 97% at thresholds of 3, 4 and 5 SD below the mean for GA, respectively (Table 4, Fig. 2G–I).

**Table 4:** Diagnostic accuracy of ultrasound measurements of head circumference for prenatal assessment of microcephaly.

Cut-off	−3 SD	−4 SD	−5 SD
Number of cohorts	2^18, 19^	2^18, 19^	2^18, 19^
Number of comparisons	45	45	45
Diagnostic odds ratio	12.7 (2.1–76.5), I^2^ = 0%	25.3 (3.7–171.6), I^2^I^2^ = 0%	48.0 (4.8–481.5), I^2^ = 0%
AUC	0.84	0.88	0.68
Sensitivity	0.84 (0.36–0.98)	0.68 (0.33–0.90)	0.58 (0.30–0.82)
Specificity	0.70 (0.34–0.91)	0.91 (0.74–0.97)	0.97 (0.83–1.00)
Positive likelihood ratio	2.6 (0.88–8.4)	7.6 (2.1–25.7)	19.3 (3.0–126.3)
Negative likelihood ratio	0.24 (0.030–1.1)	0.35 (0.11–0.76)	0.43 (0.19–0.74)
PPV (general pregnancy)	0.00075	0.00215	0.00548
NPV (general pregnancy)	0.99993	0.99990	0.99988
PPV (ZIKV-infected pregnancy)	0.0262	0.0676	0.1564
NPV (ZIKV-infected pregnancy)	0.9978	0. 9966	0.9959

Parentheses indicate 95% CI. Pre-test probabilities, i.e. incidence of microcephaly among general pregnancies and ZIKV-infected pregnancies were estimated as 0.0285% and 0.95%, respectively.

Based on these two studies, HC measurements using 3 SD below the mean had relatively high sensitivity (84%), specificity (70%), positive likelihood ratio (2.6), and negative predictive values for unspecified (99%) and ZIKV-infected pregnant populations (99%) (Table 4, Fig. 2G–I). As the SD below the mean for GA increased from 3 to 5, the sensitivity decreased while the specificity increased substantially.

Descriptive data was provided in the other six studies^13, 17, 21–24^. Among 42 fetuses prenatally diagnosed with microcephaly, Leibovitz *et al*.^21^ reported 24 true positives and 18 false positives, and a positive predictive value (PPV) of 57.1 at an HC of 3 SD below the mean for GA.

In a study of 20 suspected cases of fetal microcephaly, Stoler-Poria *et al*.^17^ confirmed five cases to be true positives and 15 false positives. The true positive cases had a HC of between 2 and 4.8 SDs below the mean for gestational age.

Wong *et al*.^22^ reported comparable z-scores for prenatal and postnatal correlations in 455 fetuses. A z-score threshold of ≤1.3 below the mean (44.6% sensitivity, 35.1% specificity, 44.9% FP rate, 45.9% FN rate,) was more sensitive and specific relative to a z-score of ≤1.7 below the mean (28.8% sensitivity, 21% specificity, 62.6% FP rate, 28.2% FN rate). Additionally, an area under the ROC curve of 0.6 suggested inaccuracy of prenatal ultrasound diagnosis of microcephaly.

One study^13^ reported a sensitivity of 85.7% and specificity of 85.3% for microcephaly detection at a HC of <5th percentile for gestational age. In this study, prenatal and postnatal findings were more consistent in the absence of coexisting brain abnormality.

In another study^24^, 11 of 16 cases of prenatally diagnosed microcephaly at a threshold of 3 SD below the mean for GA were false positive when examined at birth, giving a sensitivity of 31%. Campbell *et al*.^23^ reported the accurate identification of all ten cases of microcephaly suspected before 24 weeks gestation at the postnatal examination. There were no false positives or false negatives.

### Accuracy of ultrasound measurements of the HC to AC ratio (3 studies)

We could not perform a meta-analysis for this parameter. Descriptive information on the accuracy of ratios of the head circumference to abdominal circumference for fetal biometry assessment was provided in only three studies^18, 19, 21^.

In one study^18^, ultrasound detection of microcephaly with HC: AC ratio was consistently specific in diagnostic accuracy at all thresholds (3, 4 and 5 SDs) below the mean. For sensitivity, frequencies were lower at 5 SD (20%) and higher at 3 SD (80%), both below the mean.

Another study^19^ accurately detected the absence of microcephaly at thresholds of 3, 4 and 5 SD below the mean (specificity of 100%), with accuracy in sensitivity greatest at 3 SD (80%) below the mean. The third study^21^ identified a low sensitivity for HC: AC ratio at <5th percentile, for fetal suspicion (33.3%) and actual confirmation of microcephaly (37.5%).

### Accuracy of ultrasound measurements of BPD to FL ratio (2 studies)

A meta-analysis was not possible for this parameter. In one study^18^, the sensitivity and specificity of BPD: FL ultrasound measurements in detecting microcephaly were low at all thresholds measured (33–78%), but the specificity was high for measurements of 5 SD (87%) below the mean.

Another study^24^ noted the limitations of using the BPD: FL ratio for defining cases with or without microcephaly and reported five true positives and 11 false positives.

### Accuracy of ultrasound measurements of FL to HC ratio (3 studies)

Available studies could not be meta-analysed. In one study^18^, ultrasound measurement of FL: HC had a high sensitivity of 75–100% at 3–5 SDs and 87–100% specificity for ≤3SDs all below the mean.

Another study^19^ reported low sensitivity at 50–75% SD at all thresholds, highest at 1 SD (75%) and 85–100% specificity at ≤2 SD all below the mean for FL: HC parameter. Leibovitz *et al*.^21^ showed that at <5th percentile, an HC: FL ultrasound measurement showed a low sensitivity for both suspected (52.4%) and confirmed microcephaly (50%).

#### Risk of bias assessment and applicability concerns (QUADAS-2)

The two studies included in the meta-analysis were at a high risk of bias due to lack of pre-specified prenatal thresholds^18, 19^ and inappropriate exclusions^19^. Only one of these studies^19^ had concerns regarding applicability due to the limitation of the study population to a short interval of <2 weeks between a prenatal index scan and postnatal reference test.

In five of seven descriptive studies^13, 17, 20, 23, 24^, a high risk of bias rating was assigned. These studies limited the study population of pregnant women to the following: CMV-infection and the availability of MRI and US diagnosis^13^, Hebrew native language ability^17^, mothers who presented with phenylketonuria^20^, before 26 weeks gestation^23^ or late trimester measurements (28 to 43 weeks)^24^. The two other studies had a low risk of bias^21, 22^.

**Figure 3:**
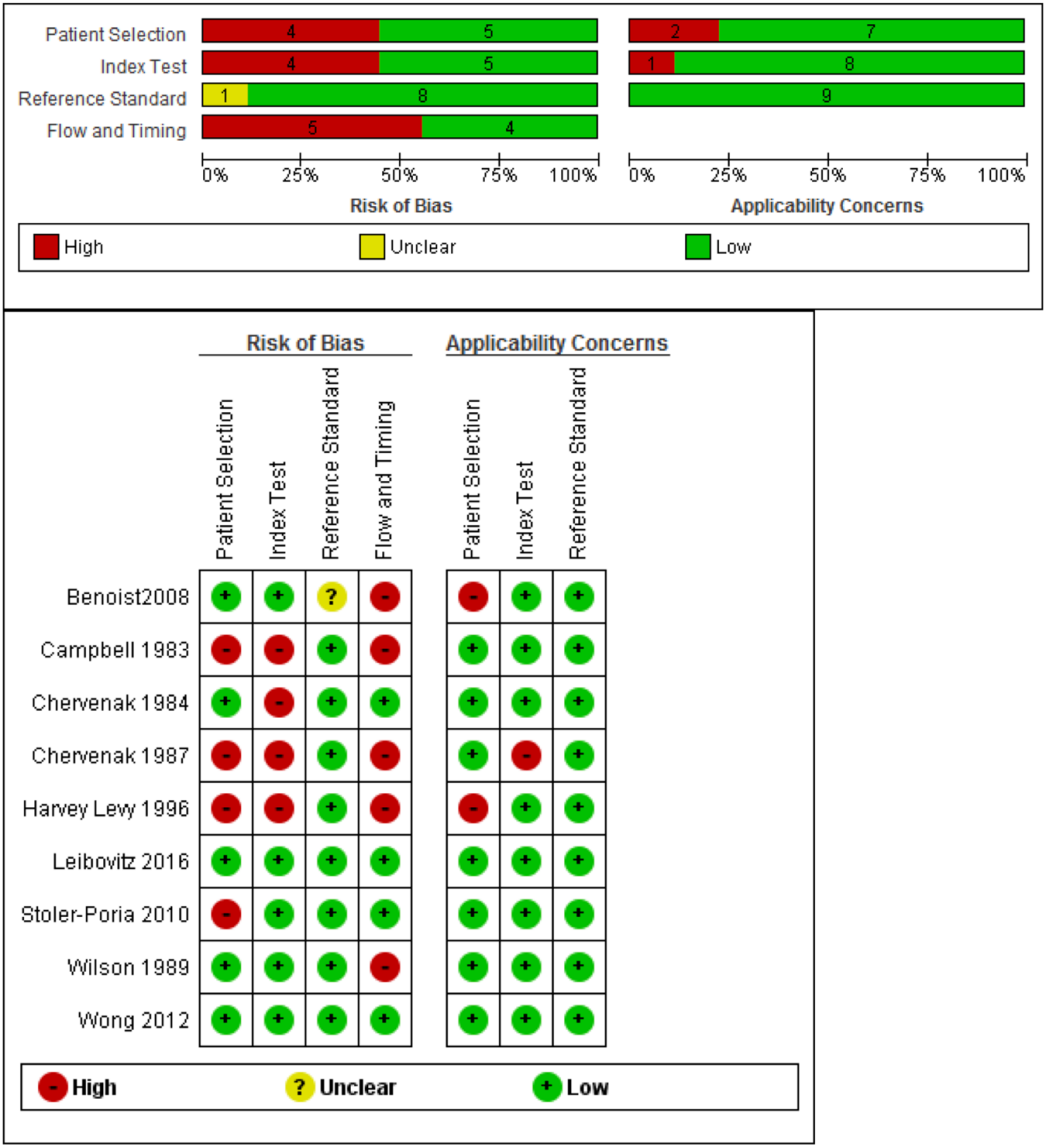
Quality Assessment of Diagnostic Accuracy Studies (QUADAS-2). Summary of risk of bias and applicability concerns of included studies.

Concerns regarding applicability were noted in two^13, 20^ of the seven studies that provided only descriptive data. These studies included only high risk mothers infected with CMV^13^ and having phenylketonuria^20^. All other five studies^17, 21–24^ had low concerns regarding applicability (Fig. 3).

## Discussion

This review provides a thorough overview of available information on the prenatal application of ultrasound for diagnosis of microcephaly. HC and OFD measurements at 4 and 5 SD below the mean had a high DOR (25.3 to 48.0) and positive likelihood ratios (7.6 to 19.3) with wide 95% confidence intervals.

Negative predictive values for unspecified- and extrapolated-ZIKV-infected pregnancies at these standard deviations were consistently high, close to 100%, although these values were derived from a relatively small number of fetuses. Thresholds of 4 and 5 SDs below the mean for OFD and HC showed a tendency to consistently “rule in” the diagnosis of fetal microcephaly with a reasonable level of confidence.

Our study indicates that the overall diagnostic test accuracy of ultrasound for predicting microcephaly at birth is limited as it varied with the applied cut-offs. Large differences were not observed among the different biometric parameters used to make a prenatal detection of microcephaly.

Ultrasound measurements of all three parameters should be recommended for cases with a high likelihood of microcephaly. Given the low incidence of microcephaly^1^, a fetal ultrasound seems not to have a large effect on the probability of identifying true cases of microcephaly.

To detect fetal microcephaly and/or brain abnormalities, the WHO currently recommends an early fetal anomaly scan between 18 to 20 weeks gestation or at the earliest possible time if after 20 weeks. A repeat ultrasound in the late second or early third trimester, usually around 28 to 30 weeks gestation^4^ is further encouraged to exclude false positives.

The inclusion of coexisting abnormalities such as intrauterine growth restriction, intracranial deformities and a detailed family history has been shown to improve the predictive value of ultrasound diagnosis^21^. Thus, setting an SD threshold to increase the accuracy of microcephaly detection in ZIKV-infected and any pregnancies should be informed by a balance of expert opinion, detailed history and analysis of other associated fetal anomalies^25^.

Variation in sensitivity and specificity for all fetal head biometric measurements (BPD, HC, OFD) observed in all studies may have been due to trimester-specific changes in fetal growth, differences in ultrasound device, techniques and patient characteristics (congenital or acquired microcephaly, the presence of other anomalies)^11, 26^. Growth appreciably slows in the third trimester in a fetus affected with microcephaly and autosomal recessive inheritance patterns may play an importantrole.

Fetuses with microcephaly are often miscarried, terminated or result in stillbirths which may explain the absence of comparative studies in ZIKV-infected pregnant women. Comparisons with postmortem or pathological samples derived from such scenarios introduce some form of bias^27^. In such cases, the estimated accuracy should be interpreted with caution.

The prenatal diagnostic accuracy of structural abnormalities affords informed maternal and health provider decisions, on whether to continue, terminate or institute fetal therapies. Potential misdiagnosis can be a source of emotional trauma during pregnancy. Hence, a review of growth standards employed and agreement with postnatal measurements can help eliminate or decrease the incidence of misdiagnosis.

To the best of our knowledge, no study at the time of conducting this systematic review had examined the variations in head measurements, in the context of microcephaly for fetuses of ZIKV-infected pregnant women. An evident lack of longitudinal or other studies indicating the best time-point for head measurements of fetuses from ZIKV-infected pregnant women is also present. Our comprehensive search strategy and lack of a date or language restrictions likely identified all studies.

Our study had limitations. Primary data was from a limited number of fetuses and reported by two studies with unclear or high risk of bias. The nature of studies included in the quantitative synthesis and an overall high risk of bias rating limit the confidence in extrapolated results for ZIKV-infected pregnant women.

Trimester-specific variation in fetal morphology visible on ultrasound measurements also restricts the use of fetal biometric parameters in isolation^28^. This proposes a need for incorporating presenting features and a detailed history of the pregnant woman. Variation in thresholds, ultrasound device and timing of assessment during pregnancy adds potential flow and timing bias.

With the influx of research on ZIKV infections in pregnancy, we acknowledge the rapid evolution of knowledge on the subject. Further studies addressing ultrasound accuracy and based on fetal biometric parameters, all relative to reference measures at birth using modern ultrasound machines will be helpful.

It is reasonable to assume that the technical improvement of ultrasound machines in the last 20 years should contribute to improved diagnostic accuracy which was lacking in the published studies published. Research on diagnostic test accuracy based on present-day ultrasound devices is needed to improve confidence in fetal microcephaly diagnosis.

In conclusion, we provide evidence for the diagnostic accuracy of ultrasound in the detection of fetal microcephaly. Ultrasound diagnostic accuracy of HC and OFD parameters at 4 and 5 SD below the mean was better at ruling in fetal microcephaly with high DOR, sensitivity, specificity and positive likelihood ratio. The relative improvement in ultrasound technology and technical skills suggests the need for new studies on the subject.

## Electronic supplementary material

Appendices to Mansucript

## References

[1] Cauchemez S (2016). Association between Zika virus and microcephaly in French Polynesia, 2013–15, a retrospective study. The Lancet 387.

[2] Oliveira Melo, A. S. *et al*. Zika virus intrauterine infection causes fetal brain abnormality and microcephaly: tip of the iceberg? *Ultrasound in Obstetrics & Gynecology* **47**, 6–7, doi:http://dx.doi.org/10.1002/uog.15831 (2016). 10.1002/uog.15831 26731034

[3] World Health Organization. *Zika situation report: Zika and potential complications* (2016).

[4] World Health Organization. *Pregnancy management in the context of Zika virus: interim guidance* (2016). 10.1016/S2214-109X(16)30098-5 27211476

[5] Benson C, Doubilet P (1991). Sonographic prediction of gestational age: accuracy of second-and third-trimester fetal measurements. AJR. American journal of roentgenology.

[6] Doubilet PM, Greenes RA (1984). Improved prediction of gestational age from fetal head measurements. AJR. American Journal of Roentgenology.

[7] Melamed, N. *et al*. Sonographic estimation of fetal head circumference: how accurate are we? *Ultrasound in Obstetrics & Gynecology* **37**, 65–71, doi:http://dx.doi.org/10.1002/uog.7760 (2011). 10.1002/uog.7760 20661958

[8] Aubry MC, Aubry JP, Dommergues M (2003). Sonographic prenatal diagnosis of central nervous system abnormalities. Childs Nervous System.

[9] Goncalves LF, Jeanty P, Piper JM (1994). The accuracy of prenatal ultrasonography in detecting congenital anomalies. American Journal of Obstetrics & Gynecology.

[10] Schwarzler P, Homfray T, Bernard JP, Bland JM, Ville Y (2003). Late onset microcephaly: failure of prenatal diagnosis. Ultrasound in Obstetrics & Gynecology.

[11] Bromley B, Benacerraf BR (1995). Difficulties in the prenatal diagnosis of microcephaly. Journal of Ultrasound in Medicine.

[12] Deter RL, Harrist RB, Hadlock FP, Carpenter RJ (1982). Fetal head and abdominal circumferences: I. Evaluation of measurement errors. Journal of Clinical Ultrasound.

[13] Benoist, G. *et al*. Cytomegalovirus-related fetal brain lesions: comparison between targeted ultrasound examination and magnetic resonance imaging. *Ultrasound in Obstetrics & Gynecology* **32**, 900–905, doi:http://dx.doi.org/10.1002/uog.6129 (2008). 10.1002/uog.6129 18991327

[14] Jones C, Athanasiou T (2005). Summary receiver characteristic curve analysis techniques in the evaluation of diagnostic tests. Annals Thorac Surg.

[15] Reitsma, H. *et al*. *9 Assessing Methodological Quality* (2009).

[16] Whiting P (2003). The development of QUADAS: a tool for the quality assessment of studies of diagnostic accuracy included in systematic reviews. BMC medical research methodology.

[17] Stoler-Poria S, Lev D, Schweiger A, Lerman-Sagie T, Malinger G (2010). Developmental outcome of isolated fetal microcephaly. Ultrasound in obstetrics & gynecology: the official journal of the International Society of Ultrasound in Obstetrics and Gynecology.

[18] Chervenak FA, Jeanty P, Cantraine F (1984). The diagnosis of fetal microcephaly. American journal of obstetrics and gynecology.

[19] Chervenak FA, Rosenberg J, Brightman RC, Chitkara U, Jeanty P (1987). A prospective study of the accuracy of ultrasound in predicting fetal microcephaly. Obstetrics & Gynecology.

[20] Levy HL, Lobbregt D, Platt LD, Benacerraf BR (1996). Fetal ultrasonography in maternal PKU. Prenatal Diagnosis.

[21] Leibovitz Z (2015). Microcephaly at birth‐the accuracy of three references for fetal head circumference. How can we improve prediction? Ultrasound in Obstetrics & Gynecology.

[22] Wong J, Howe C, Bianco A, Green R, Stone J (2012). The accuracy of prenatal ultrasound in the diagnosis of true microcephaly. American journal of obstetrics and gynecology.

[23] Campbell S, Pearce JM (1983). The prenatal diagnosis of fetal structural anomalies by ultrasound. Clinics in Obstetrics & Gynaecology.

[24] Wilson RD, Hitchman D, Wittman BK (1989). Clinical follow-up of prenatally diagnosed isolated ventriculomegaly, microcephaly and encephalocele. Fetal Therapy.

[25] Malinger G (2002). A normal second-trimester ultrasound does not exclude intracranial structural pathology. Ultrasound in Obstetrics & Gynecology.

[26] Mandell J, Bromley B, Peters CA, Benacerraf BR (1995). Prenatal sonographic detection of genital malformations. Journal of Urology.

[27] Adcock LM, Moore PJ, Schlesinger AE, Armstrong DL (1998). Correlation of ultrasound with postmortem neuropathologic studies in neonates. Pediatric Neurology.

[28] Grannum P, Pilu G (1987). In utero neurosonography: the normal fetus and variations in cranial size. Seminars in Perinatology.

