# Supplementary material for: Diagnostic Accuracy of Ultrasound Scanning for Prenatal Microcephaly in the context of Zika Virus Infection: A Systematic Review and Meta-analysis
Source: Sci Rep. 2017 May 23;7:2310. doi: 10.1038/s41598-017-01991-y (PMC5442132; doi:10.1038/s41598-017-01991-y)
Supplement: Supplementary file 1 — Appendices to Mansucript [file 41598_2017_1991_MOESM1_ESM.pdf]

Diagnostic Accuracy of Ultrasound Scanning for Prenatal Microcephaly in the context of Zika Virus Infection: A Systematic Review and Meta-analysis

Ezinne C Chibueze,[a] Alex JQ Parsons,[b] Katharina da Silva Lopes,[a] Takemoto Yo,[a] Toshiyuki Swa,[a, c] Chie Nagata,[d] Nobuyuki Horita,[e] Naho Morisaki,[f] Olukunmi O Balogun,[a] Amarjargal Dagvadorj,[a, g] Erika Ota,[a, h] Rintaro Mori,[a] Olufemi T Oladapo [i]

[a] Department of Health Policy, National Center for Child Health and Development, Tokyo, Japan

[b] Department of Global Health Policy, Graduate School of Medicine, University of Tokyo, Tokyo, Japan

[c] Graduate School of Human Sciences, Osaka University, Osaka, Japan

[d] Department of Education for Clinical Research, National Center for Child Health and Development, Tokyo Japan

[e] Pulmonology, Graduate School of Medicine, Yokohama City University, Yokohama, Japan

[f] Department of Social Medicine, National Center for Child Health and Development, Tokyo, Japan

[g] Department of Health Informatics, Kyoto University, Yoshida Konoe-cho, Syako-ku, Kyoto, Japan

[h] St. Luke's International University, Graduate school of Nursing, Global Health Nursing, Tokyo, Japan

[i] UNDP/UNFPA/UNICEF/WHO/World Bank Special Programme of Research, Development and Research Training in Human Reproduction (HRP), Department of Reproductive Health and Research World Health Organization, Geneva, Switzerland.

Appendix 1 : Database search strategies for prenatal diagnosis of microcephaly in the context of ZIKV infection on March 3rd 2016

No	MEDLINE	Results
1	exp Flavivirus/	16541
2	exp Flavivirus Infections/	20393
3	(zika or flavivi* or "flavi vi*" or dengue or (encephalitis adj3 (japan* or "st louis" or "tick borne")) or "west nile fever*" or "yellow fever*").mp.	34755
4	or/1-3	35485
5	Chikungunya Virus/	1534
6	Chikungunya Fever/	584
7	chikungunya.mp.	2689
8	Arboviruses/	3169
9	arbovirus*.mp.	8293
10	or/5-9	10400
11	or/4,10	42307
12	exp "Congenital, Hereditary, and Neonatal Diseases and Abnormalities"/	1E+06
13	exp "Embryonic and Fetal Development"/	229585
14	exp Embryonic Structures/	391915
15	exp Nerve Growth Factors/	41137
16	Cephalometry/	24107
17	Maternal Exposure/	6172
18	exp Pregnancy/	784084
19	Pregnant Women/	
20	Prenatal Care/	21837
21	exp Prenatal Diagnosis/	63551
22	or/12-21	2E+06
23	11 and 22	1607
24	(microcephal* or microlissencephal* or anencephal* or ((congenital or brain or cerebral or "white matter" or nerv* or neur*) adj3 (malformation* or abnormalit* or defect* or calcification or development* or growth))).mp.	266351
25	(cephalometr* or (head adj3 (circumference* or size))).mp.	31460
26	(prenatal or antenatal or fetus or fetal or foetus or foetal or gestation* or intrauter* or pregnan* or "expectant mother*").mp.	1E+06
27	embryo*.mp.	376522
28	stillbirth*.mp.	10082
29	neurotroph*.mp.	34460

30	or/24-29	2E+06
31	11 and 30	1811
32	31 not medline.st.	124
33	exp Nervous System Malformations/cn, em, ep, et [Congenital, Embryology, Epidemiology, Etiology]	7727
34	TORCH.mp.	930
35	Toxoplasma/	11353
36	exp Toxoplasmosis/	17866
37	exp Viruses/	673651
38	exp Virus Diseases/	783448
39	Aedes/	11697
40	or/34-39	1E+06
41	33 and 40	243
42	or/23,32,41	1959
43	remove duplicates from 42	1954
44	43 not (animals.sh. not (humans.sh. or 39))	1216
45	limit 44 to (comment or editorial or news or newspaper article)	45
46	44 not 45	1171

No.	EMBASE	Results
#1	'flavivirus'/exp	23321
#2	'flavivirus infection'/exp	22456
#3	zika OR flavivi* OR 'flavi vi*' OR dengue OR encephalitis NEAR/3 (japan* OR 'st louis' OR 'tick borne') OR 'west nile fever*' OR 'yellow fever*'	40882
#4	'chikungunya virus'/de	2271
#5	'chikungunya'/de	1519
#6	chikungunya	3625
#7	'arbovirus'/de	4180
#8	arbovirus*	7688
#9	#1 OR #2 OR #3 OR #4 OR #5 OR #6 OR #7 OR #8	50455
#10	'congenital malformation'/exp	713508
#11	'prenatal development'/exp	201031
#12	'embryo (anatomy)'/exp	213844
#13	'neurotrophic factor'/exp	60485
#14	'cephalometry'/de	20598
#15	'pregnancy'/exp	649044
#16	'expectant mother'/de	288

#17	'prenatal care'/exp	120829
#18	'prenatal diagnosis'/exp	87727
#19	microcephal* OR microlissencephal* OR anencephal* OR (congenital OR brain OR cerebral OR 'white matter' OR nerv* OR neur*) NEAR/3 (malformation* OR abnormalit* OR defect* OR calcification OR development* OR growth)	535932
#20	cephalometr* OR head NEAR/3 (circumference* OR size)	33737
#21	prenatal OR antenatal OR fetus OR fetal OR foetus OR foetal OR gestation* OR intrauter* OR pregnan* OR 'expectant mother*' OR stillbirth*	1212384
#22	embryo*	515358
#23	neurotroph*	53990
#24	#10 OR #11 OR #12 OR #13 OR #14 OR #15 OR #16 OR #17 OR #18 OR #19 OR #20 OR #21 OR #22 OR #23	2536003
#25	#9 AND #24	2579
#26	'nervous system malformation'/exp/'congenital disorder','etiology','epidemiology'	18065
#27	torch	1438
#28	'toxoplasma'/exp	16001
#29	'toxoplasmosis'/de	19821
#30	'virus'/exp	893164
#31	'virus infection'/exp	964171
#32	'aedes'/exp	13176
#33	#27 OR #28 OR #29 OR #30 OR #31 OR #32	1492147
#34	#26 AND #33	513
#35	#25 OR #34	3081
#36	#35 AND [humans]/lim NOT ([animals]/lim NOT #32)	1307

**No**	**CENTRAL**	**Results**
#1	MeSH descriptor: [Flavivirus] explode all trees	124
#2	MeSH descriptor: [Flavivirus Infections] explode all trees	241
#3	zika or flavivi* or "flavi vi*" or dengue or (encephalitis near/3 (japan* or "st louis" or "tick borne")) or "west nile fever*" or "yellow fever*":ti,ab,kw	536
#4	#1 or #2 or #3	536
#5	MeSH descriptor: [Chikungunya virus] this term only	4
#6	MeSH descriptor: [Chikungunya Fever] this term only	2
#7	chikungunya:ti,ab,kw	14
#8	MeSH descriptor: [Arboviruses] this term only	1

#9	arbovirus*:ti,ab,kw	6
#10	#5 or #6 or #7 or #8 or #9	20
#11	#4 or #10	553
#12	MeSH descriptor: [Congenital, Hereditary, and Neonatal Diseases and Abnormalities] explode all trees	15296
#13	MeSH descriptor: [Embryonic and Fetal Development] explode all trees	3554
#14	MeSH descriptor: [Embryonic Structures] explode all trees	2655
#15	MeSH descriptor: [Cephalometry] this term only	592
#16	MeSH descriptor: [Maternal Exposure] this term only	46
#17	MeSH descriptor: [Prenatal Care] this term only	1197
#18	MeSH descriptor: [Prenatal Diagnosis] explode all trees	1021
#19	(microcephal* or microlissencephal* or anencephal* or ((congenital or brain or cerebral or "white matter" or nerv* or neur*) near/3 (malformation* or abnormalit* or defect* or calcification or development* or growth))):ti,ab,kw	3633
#20	(cephalometr* or (head near/3 (circumference* or size))):ti,ab,kw	1385
#21	(prenatal or antenatal or fetus or fetal or foetus or foetal or gestation* or intrauter* or pregnan* or "expectant mother*" or stillbirth*):ti,ab,kw	37768
#22	embryo*:ti,ab,kw	3817
#23	neurotroph*:ti,ab,kw	587
#24	#12 or #13 or #14 or #15 or #16 or #17 or #18 or #19 or #20 or #21 or #22 or #23	55078
#25	#11 and #24	13
#26	MeSH descriptor: [Nervous System Malformations] explode all trees and with qualifier(s): [Etiology - ET]	9
#27	#25 or #26	22

**Search ID#**	**CINAHL**	**Results**
S34	S26 OR S33	200
S33	S27 AND S32	15
S32	S28 OR S29 OR S30 OR S31	145712
S31	(MH "Virus Diseases+")	137287
S30	(MH "Viruses+")	29364
S29	(MH "Toxoplasmosis+")	875
S28	TORCH	261
S27	(MH "Nervous System Abnormalities+/EM/EP/ET")	781
S26	S11 AND S25	185

S25	S12 OR S13 OR S14 OR S15 OR S16 OR S17 OR S18 OR S19 OR S20 OR S21 OR S22 OR S23 OR S24	382515
S24	(prenatal or antenatal or fetus or fetal or foetus or foetal or gestation* or intrauter* or pregnan* or "expectant mother*" or stillbirth*)	176976
S23	(cephalometr* or (head N3 (circumference* or size)))	4217
S22	(microcephal* or microlissencephal* or anencephal* or ((congenital or brain or cerebral or "white matter" or nerv* or neur*) N3 (malformation* or abnormalit* or defect* or calcification or development* or growth)) or neurotroph*)	24351
S21	(MH "Prenatal Diagnosis+")	12212
S20	(MH "Prenatal Care") OR (MH "Prepregnancy Care")	11885
S19	(MH "Expectant Mothers")	2757
S18	(MH "Pregnancy+")	138371
S17	(MH "Maternal Exposure")	1376
S16	(MH "Cephalometry")	2846
S15	(MH "Nerve Growth Factors+")	856
S14	(MH "Embryonic Structures+")	27678
S13	(MH "Growth and Embryonic Development+")	117358
S12	(MH "Congenital, Hereditary, and Neonatal Diseases and Abnormalities+")	121383
S11	S4 OR S10	4220
S10	S5 OR S6 OR S7 OR S8 OR S9	844
S9	arbovirus*	485
S8	(MH "Arboviruses")	44
S7	chikungunya	382
S6	(MH "Chikungunya Fever")	198
S5	(MH "Chikungunya Virus")	144
S4	S1 or S2 or S3	3798
S3	(zika or flavivi* or "flavi vi*" or dengue or (encephalitis N3 (japan* or "st louis" or "tick borne")) or "west nile fever*" or "yellow fever*")	3763
S2	(MH "Flavivirus Infections+")	2983
S1	(MH "Flavivirus")	45

Appendix 2: List of excluded studies and reasons for exclusion

No	Study	Title	Country	Report type (non-English, conference abstract)	Setting (e.g. facility, medical record)	Reason for exclusion
1	Alarcon 2013	Neuroimaging findings as predictors of neurodevelopmental outcome in patients with symptomatic congenital cytomegalovirus (CMV)	Spain	Journal Article	Hospital	No data relevant to review objective. The aim of the retrospective study was to investigate clinical, CSF, and neuroimaging findings in the newborn period as predictors of long-term neurodevelopmental outcome in patients with symptomatic congenital CMV infection. Neurologic examination was used to assess neurodevelopment
2	Braddock 2014	35th Annual David W Smith Workshop on Malformations and Morphogenesis: Abstracts of the 2014 Annual Meeting	USA	Meeting Abstracts	NA	No data relevant to review objective. No relevant abstract within
3	Abdel Aziz e-poster 2014	Fetal medicine conference	UK	Poster	Hospital	No postnatal comparisons. Fetal anomalies were detected. Microcephaly or related anomalies were unnamed
4	Abdella Rana	Sonographic evaluation of fetal abdominal circumference and cerebro-placental Doppler indices for the prediction of fetal macrosomia in full term pregnant women. Cohort study	Egypt	Journal Article	Hospital	No data relevant to review objective. No microcephaly and postnatal comparisons
5	Chen CP 2011	Galloway-Mowat syndrome: Prenatal ultrasound and perinatal magnetic resonance imaging findings	Taiwan	Case Report	Hospital	No data relevant to review objective. Postnatal comparison was to MRI. A single case report
6	Amini 2010	The clinical impact of fetal magnetic resonance imaging on	Sweden	Journal Article	Hospital	No data relevant to review objective. Prenatal comparison to postnatal, but absence of microcephaly among the analysed anomalies (rather Dandy walker and holoprosencephaly)

		management of central nervous system (CNS) anomalies in the second trimester of pregnancy				
7	Adcock 1998	Correlation of Ultrasound With Postmortem Neuropathologic Studies in Neonates	USA	Journal Article	Hospital	No data relevant to review objective. Prenatal and postmortem comparisons described but microcephaly was not one of the neurodiagnosis
8	Altman 1997	New charts for ultrasound dating of pregnancy	London	Journal Article	Hospital	No data relevant to review objective. Antenatal measurements were correlated to gestational age during pregnancy
9	Amer 2014	Foetal central nervous system anomalies: Frequency and feto-maternal outcome	Saudi Arabia	Journal Article	Hospital	No data relevant to review objective and lacks details on fetal biometric parameters assessed. A retrospective descriptive study where antenatal and postnatal outcomes were reported with no details on measured parameters
10	Briceno 2013	Fetal Size Charts for a Population From Cali, Colombia	USA	Journal Article	Hospital	No data relevant to review objective. Antenatal ultrasound measurements of non-microcephalic fetuses correlated to fetal size all during pregnancy
11	Briceno 2013	Charts for Fetal Age Assessment Based on Fetal Sonographic Biometry in a Population From Cali, Colombia	USA	Journal Article	Hospital	No data relevant to review objective, no postnatal comparison or mention of microcephaly. Antenatal ultrasound measurements correlated to fetal size all during pregnancy, no postnatal measurements
12	Go 2001	Dutch article	Amster dam	Journal Article	Hospital	No data relevant to review objective. Antenatal ultrasound comparisons to postnatal Ultrafast MRI, no case of microcephaly
13	Jozsef 2011	Hungarian article	Budape st	Journal Article	Hospital	No data relevant to review objective. Review paper
14	Ozkan 2011	Our clinical experience about prenatal diagnosis and neonatal outcomes of fetal central nervous system anomalies	Turkey	Journal Article	Hospital	No prenatal comparison. A retrospective study in which one of the patients had microcephaly. Parameter for detection was fetal head size of ≥2 SD below the mean
15	Duncan 2005	A comparison of fetal organ measurements by	UK	Journal Article	Hospital	No data relevant to review objective. Study was aimed at determining fetal growth restriction, ultrasound and

		echo-planar magnetic resonance imaging and ultrasound				MRI measurements of fetal organ size in normal and fetal growth-restricted pregnancies, to determine whether there are any differences in fetal organ size detected by the two techniques all at prenatal stage. Microcephaly was not mentioned
16	Debost Legrande 2014	False positive morphologic diagnoses at the anomaly scan: marginal or real problem,	France	Journal Article	Hospital	No postnatal comparisons. Broad cohort of fetal malformations with general TP, TN, FP, FN. Mothers with a prenatal diagnosis of fetal malformation were included in the study, though unnamed (no mention of microcephaly). All measurements were prenatal
17	Deter 1982	Fetal Head and Abdominal Circumferences: Evaluation of Measurement Errors	USA	Journal Article	Hospital	No data relevant to review objective. In this retrospective study, prenatal and postnatal measurements of fetal head and abdominal circumference were compared in mothers who gave birth via cesarean and vaginal delivery and their fetus
18	Karp 1974	Use of Ultrasound in the prenatal exclusion of primary microcephaly	USA	Journal Article	Hospital	No data relevant to review objective. Case report with only one case of suspected microcephaly in a 3rd pregnancy due to a familial history of microcephaly. The mother who eventually gave birth to a normal head sized baby
20	Chalana 1996	Automatic fetal head measurements from sonographic images	USA	Journal Article	Center for Bioengineerin g	No data relevant to review objective. The aim of the study was to evaluate the performance of the image processing technique by comparing the resulting measurements with those made by experienced sonographers, not relevant to microcephaly
21	Den Hollander 2000	Congenital microcephaly detected by prenatal ultrasound: genetic aspects and clinical significance	Netherl ands	Journal Article	Hospital	Unclear parameters and not in line with review objective. Prenatal and postnatal comparisons were limited to fetuses with microcephaly defined according to Snijders reference charts
22	Pilu 1998	Prenatal diagnosis of microcephaly assisted by vaginal sonography and power Doppler	Italy	Journal Article	Hospital	No postnatal comparisons and fetal biometric parameters. Case report on two cases
23	Hadlock 1984	The femur	USA	Journal Article	Hospital	No postnatal comparisons

		length/head circumference relation in obstetric sonography				
24	Hagmann 2011	Cerebral measurements made using cranial ultrasound in term Ugandan newborns	Uganda	Journal Article	Hospital	No prenatal comparisons. Cranial Ultrasound in newborn infants, no measurement before birth
25	Krishnamoorthy 2011	Early Cranial Ultrasound Lesions Predict Microcephaly at Age 2 Years in Preterm Infants	USA	Journal Article	14 institutions in 11 cities in 5 US states	Preterm birth comparisons. Only microcephalic preterm (<28 weeks) babies. Head circumference was measured at preterm birth and at 24 months post-term equivalent. Infants had MCP at 2 years but not at birth. Study reported high negative predictive values (91% and 90%, respectively) for microcephaly. 6% of children who had a normal ultrasound scan were microcephalic
26	Kuban 2009	Developmental Correlates of Head Circumference at Birth and Two Years in a Cohort of Extremely Low Gestational Age Newborns	USA	Journal Article	14 institutions in 11 cities in 5 US states	Preterm birth comparisons. Head circumference was measured at birth and at 24 months post-term equivalent
27	Sarno 2016	Zika Virus Infection and Stillbirths: A Case of Hydrops Fetalis, Hydranencephaly and Fetal Demise	Brazil	Journal Article	Hospital	No data relevant to review objective
28	Stashinko 2004	A Retrospective Survey of Perinatal Risk Factors of 104 Living Children With Holoprosencephal y	USA	Journal Article	3 Carter Centers for Brain Research in Holoprosence phaly and Related Brain Malformations , database study	No data relevant to review objective. No separate data for children eventually confirmed as having microcephaly at birth, only holoprosencephaly
29	Tolmie 1987	Microcephaly: Genetic counselling and antenatal	UK	Journal Article	Institute of medical genetics	Case series

		diagnosis after the birth on a affected child				
30	Vezina 2004	Congenital Malformations of the Brain: Prenatal and Postnatal Imaging		Journal Article		No data relevant to review objective. A review
31	Bergsjo 1975	Observer error in ultrasonic fetal cephalometry	Norwa y	Journal Article		No data relevant to review objective. Focus of the paper was to investigate observer error in fetal cephalometry, no mention about microcephaly, A study narratively reporting on a personal experience of prenatal diagnosis of structural fetal anomalies
32	Karl 2011	Fetale Neurosonografie: die erweiterte Untersuchung des ZNS beim Fetus	Germa ny	Journal Article		No data relevant to review objective. Educational paper about ultrasound examination of the fetal CNS for diagnostic purposes
33	Kleber de Oliveira 2016	Increase in Reported Prevalence of Microcephaly in Infants Born to Women Living in Areas with Confirmed Zika Virus Transmission During the First Trimester of Pregnancy — Brazil, 2015	Brazil	Report		No data relevant to review objective.. Overview of the situation of microcephaly and Zika virus infection in Brazil in 2015
34	Elsayed 2013	Antenatal Ultrasound Diagnosis of Fetal Anomalies at a University Hospital	Kuwait	Journal Article	Hospital	No data on fetal biometric parameters used. The aim of the present study was to highlight the role of antenatal ultrasound in detection of fetal anomalies. Although nine microcephalic cases were included, there is no further information on how they were diagnosed
35	Gardosi 2002	Ultrasound biometry and fetal growth restriction		Journal Article	Review	No data relevant to review objective. Review of FGR and SGA
36	Ioannou 2012	Systematic review of methodology use in ultrasound studies aimed at created charts of fetal size		Journal Article	Review	No data relevant to review objective. A review of multiple population based US fetal growth charts, but no mention of how to diagnose microcephalus

37	Nazir 2013	Detection of Congenital Malformations in Healthy Pregnant Ladies Undergoing Routine Scan	Pakistan	Journal Article	Hospital	No data relevant to review objective. 1 case of microcephaly with no further information. Paper mentions detection rate and false positive rate of screening at 18-20 weeks for congenital anomalies but not microcephaly explicitly
38	Pilu 1986	Antenatal recognition of cerebral anomalies	Italy	Journal Article	review	No data relevant to review objective. A review
39	Salamanca 1992	Prenatal ultrasound semiography of anencephaly	Spain	Journal Article	Hospital	No data relevant to review objective. Case series on ancephaly. Mentions diagnostic rate of ancephaly but not microcephaly
40	Sarti 1981	Correlation of Biparietal and Fetal Body Diameters	USA	Journal Article	Hospital	A case report, describes five cases in which one was diagnosed as MCP and confirmed at birth using the ratio of BPD and abdominal measurement parameters
41	Schlensker 1989	Antenatal Diagnosis of Neural Tube Defects	Germany	Journal Article	article in German	No data relevant to review objective. Paper on only fetuses with neural tube defect. Based on the English translation, the sonographic diagnosis was made in 11 cases without additional gap formation of the brain cranium due to more than 3 standard deviations below the normal. There were serious additional malformations. All children were born vaginally between the 28th and 38th week
42	Thomas 2016	Local Transmission of Zika virus	Puerto Rico	Weekly report	National surveillance	No data relevant to review objective. Paper on 155 suspected cases of Zika. No numbers on how many were pregnant women
43	Issel 1975	The measurement of fetal growth during pregnancy by ultrasound (B-Scan)	Germany	Journal Article	Hospital	No postnatal comparisons
44	Johnsen 2005	Fetal age assessment based on femur length at 10–25 weeks of gestation, and reference ranges for femur	Norway	Journal Article	Hospital	No postnatal comparisons

		length to head circumference ratios				
45	Schuler-Faccini 2016 (Lavania filename)	Possible Association Between Zika Virus Infection and Microcephaly -- Brazil, 2015. MMWR: Morbidity & Mortality Weekly Report, 2016	Brazil	Weekly report	National	No data relevant to review objective. No systematic experimental structure, data on ultrasound index not interpretable in the context of our review
46	Leray 2004	Late onset microcephaly: failure of prenatal diagnosis	France	Case report	Hospital	No data relevant to review objective. educational paper about ultrasound examination of the fetal CNS for diagnostic purposes
47	Levaillant 2008	Fetal sphenoid bone: imaging using three-dimensional ultrasound and computed tomography	France	Picture of the Month	Hospital	No data relevant to review objective. No comparisons
48	Oliviera 2016	Zika virus intrauterine infection causes fetal brain abnormality and microcephaly: tip of the iceberg?	Brazil	Case report	National	No data relevant to review objective. Prenatal biometric values for two children, along with other general info on the current situation in the Narrative report
49	Blondin 2008	Foreign article			Hospital	No data relevant to review objective. The authors present results from MRI which they did after US to confirm the first diagnosis. No information on the use of any measurements after birth
50	Vimercati 1999	The diagnostic role of “in utero” magnetic resonance imaging	Italy	Retrospective cohort study	Hospital	No primary data relevant to our objective
51	D Addario 2003	Editorial fetal neurosonology	NA	Editorial		No data relevant to review objective. An Editorial
52	Warsof 1986	Routine ultrasound for antenatal detection of intrauterine growth retardation	USA	Journal Article	Hospital	No postnatal comparisons
53	Wald 2003	Verification of	Austria	Journal Article	Hospital	No data relevant to review objective.

		Anomalies of the Central Nervous System Detected by Prenatal Ultrasound				It was mentioned that in 29 live births, hydrocephaly, meningomyelocele, and microcephaly had always been correctly identified prenatally, though MCP cases were not given separately. The accuracy of fetal ultrasound (US) in diagnosing CNS malformations was assessed to determine reliability for medical indication for abortions without resorting to magnetic resonance imaging (MRI)
54	Xu Huixiong, Zhang Qingping, Xiao Xiantao, Wen Liangzhen, Xu Jianping, Chen Xinlin	A Preliminary Clinical Study of Three-Dimensional Ultrasonography in Prenatal Diagnosis	China	Journal Article	Hospital	No postnatal comparison. The study evaluated the clinical value of three-dimensional ultrasonography (3DUS) in prenatal diagnosis and compared with 2DUS
55	Susan Campbell Westerway, Alastair Davison and Simon Cowell	Ultrasonic fetal measurements: new Australian standards for the new millennium	Australia	Journal Article	Hospital	No postnatal comparison. Includes prenatal OFD and BPD, though the aim of the study was to establish normal growth curves
56	Gretchen Vogel	A race to explain Brazil’s spike in birth defects	Brazil	News article		No data relevant to review objective. A news report
57	Viora E	Prenatal diagnosis of fetal: microcephaly contribution of power Doppler	Italy	Journal Article	Hospital	Unclear on the fetal biometric parameter used. A case report
58	Carolyn Miller, Wolfgang Losken, Richard Towbin, A’delbert Bowen, Mark P. Mooney, Alex Towbin, Richard S. Faix	Ultrasound Diagnosis of Craniosynostosis	USA	Journal Article	Hospital records/case series	No data relevant to review objective. Study focused on craniosynostosis
59	Roosecelis Brasil Martines, Julu Bhatnagar, M. Kelly Keating, Luciana Silva-Flannery, Atis Muehlenbachs	Evidence of Zika Virus Infection in Brain and Placental Tissues from Two Congenitally Infected Newborns and Two Fetal Losses — Brazil, 2015	Brazil	Report	Records	No data relevant to review objective. Article is a report suggesting/implicating Zika virus infection in samples from two newborns (born at 36 and 38 weeks gestation) with microcephaly who died within 20 hours of birth and two miscarriages

60	Zlotogora 2010	The impact of Prenatal diagnosis and termination of Pregnancy on the relative incidence of malformations at Birth among Jews and Muslim Arabs in Israel*	Israel	Journal Article	MOH Database	No data relevant to review objective. The study aimed to determine the impact of prenatal diagnosis and pregnancy termination on the relative incidence of malformations at birth among Jews and Muslim Arabs in Israel
61	Joo 2008	Craniospinal malformations in a twelve-year feto-pathological study; the efficiency of ultrasonography in view of feto-pathological investigations	Hungar y	Journal Article	Hospital	No data relevant to review objective
62	Ventura 2016	Ophthalmological findings in infants with microcephaly and presumable intra-uterus Zika virus infection	Brazil	Journal Article	Hospital	No data relevant to review objective. Only described ophthalmological measurements in the eyes in Zika infected infants with microcephaly
63	Bhushan	The reliability of neonatal head circumference measurement	USA	Journal article	Hospital	No data relevant to review objective. No prenatal comparisons
64	Borrell	Clinical Value of the 11- to 13þ6-Week Sonogram for Detection of Congenital Malformations: A Review	Spain	Journal article	Hospital	No data relevant to review objective. A Review
65	Karp 1974	Use of ultrasound in the prenatal exclusion of primary microcephaly	USA	Journal article	Hospital	A case report
66	Kurniawan	Predicting head circumference at birth: a study in a Dutch population using the Rosavik growth model	Netherl and	Journal article	Hospital	Study aimed at predicting head circumference relative to the Rosavik growth model
67	Kurtz 1980	Ultrasound criteria for in utero diagnosis of	USA	Journal article	Hospital	Unclear fetal biometric parameter assessed. A retrospective study aimed at analysing prenatally diagnosed

		microcephaly				microcephalic fetuses and their associated anomalies
68	Lenke 1983	Ultrasongraphic failure of early detection of fetal microcephaly in maternal phenylketonuria	USA	Journal Article		No information on fetal biometric parameter assessed. A case report
69	Wang 2008	The clinical value of a cross-sectional view of the fetal brain in screening for major brain malformations at 11–14 weeks		Meeting Abstract	Hospital	No data relevant to review objective. Cerebral malformations diagnosed at 11-14 weeks, but not microcephaly. Only holoprosencephaly and acrania
70	Westerway 2006	Ultrasonic fetal measurements: new Australian standards for the new millennium	Austral ia	Journal Article	Hospital	No postnatal comparison, no microcephaly
71	Alvarez 1984	French article	Belgiu m	Journal Article	Hospital	No postnatal comparison. Fetal anomalies were detected. Microcephaly and related anomalies were named
72	Campbell 1985	Early Diagnosis of Fetal structure abnormalities	Englan d	Journal Article	Hospital	No data relevant to review objective. Narrative on a personal experience at a hospital where nine cases of microcephaly have been accurately diagnosed before the 26th week of pregnancy and diagnosed at birth (mentioned the use of head to abdomen ratio to be of value)
73	Chen CP 1992	Early diagnosis of Fetal acrania	China	Journal Article	Hospital	No data relevant to review objective
74	Chen CP 2006	Recurrent Galloway-Mowat syndrome associated with abnormal prenatal sonographic findings	China	Journal Article	Hospital	No data relevant to review objective. Case report on a syndrome which presents with microcephaly as one of its features
75	Chen CP 2007	Perinatal imaging findings of Galloway-Mowat Syndrome	China	Journal Article	Hospital	No data relevant to review objective. Case report on image findings of a syndrome which presents with microcephaly as one of its features
76	Goldstein 1988	Sonographic assessment of the fetal frontal lobe: A potential tool for prenatal	Italy	Journal Article	Hospital	No data relevant to review objective. No postnatal measurements are available. The presence of microcephaly was estimated using the frontal lobe distance

		diagnosis of microcephaly				
77	Greco 1987	Detailed Ultrasound as a screening method for craniospinal abnormalities	Italy	Journal Article	Multicenter Hospital records of 16 hospitals	No information on fetal biometric parameter assessed. In this retrospective study routine scans were conducted on 9325 mothers, this included 8930 routine scans and 395 mothers at high risk of giving birth to craniospinal defective babies to assess gestational age and rule out structural defects. Mothers were measured at early 2nd trimester 18-22 weeks GA. 5 FN cases of MCP were detected
78	Grannum 1987	In utero neurosonography: the normal fetus and variations in size	USA	Journal Article	Not specified	No data relevant to review objective. Based on a model, predicted BPD tables are available, no postnatal measurements or relation to abnormal head size or other measurements for abnormalities
79	Holhler 1981	Comparison of ultrasound femur length and BPD in late pregnancy	USA	journal Article	Not specified	No postnatal comparisons. Study showed a linear relationship between fetal BPD and fetal femur with US measurements done in the antenatal period at 23-40 gestational weeks
80	Le Guern 1984	French article	France	Journal Article	Not specified	No data relevant to review objective. A review on brain sonography
81	Mlakar 2016	Zika virus associated with microcephaly	Sloveni a	Journal Article	Hospital	No data relevant to review objective. A case report. In which the pregnancy was terminated at 32 weeks and confirmed to have prominent microcephaly
82	Zhou 2004	Significance of ultrasonic diagnosis in fetuses with deformities of central nervous system	USA	Journal Article	Hospital records	No data relevant to review objective
83	De la Calle 2014	The value of prenatal brain MRI and US in the management of congenital CMV infection	Spain	Poster	Hospital referral	No information on fetal biometric parameter assessed. Uninterpretable data. Review of prenatal ultrasound and MRI in ten cases with two cases of prenatally diagnosed microcephaly and compared with transfontanellar ultrasound exam and follow-up at 18 months
84	Kirkinen et al 1979	Foreign language publication (Finnish)	NA	NA	NA	No postnatal comparisons. Prenatal ultrasonography only
85	Mateeva E 1983	Foreign language publication	Bulgari a	Case Report	NA	No data relevant to review objective

		(Slavic)				
86	Parant et al	Antenatal Diagnosis of Holoprosencephal y: a series of 12 cases	France	Case Series		No information on fetal biometric parameter assessed. 63% of cases were microcephaly, BPD measurements available
87	Prenzlau 1977	German Article	Germa ny	Review article	NA	No primary data. They describe the method of detection of fetal malformation
88	Se Jean 1983	French Article	France	Review Article	Review	No data relevant to review objective. A review
89	Litschgi 1986	German Article	Germa ny	Case reports	NA	No data relevant to review objective. Method of confirmation of fetal abnormality is unclear. Antenatal US as early as 15 - 32 weeks enabled detection of three microcephalic fetuses and early decision on TOP. In the 4th patient, spontaneous birth occurred at term and US had not been employed in this patient the patients had not been monographed
90	Hinselman 1983	German Article	Germa ny	Journal Article	Hospital	No data relevant to review objective. Study on several cases. No case of microcephaly
91	Hong 2004	The value of ultrasonography in prenatal diagnosis of congenital defects (Vietnamese)	Vietna m	Journal Article	Hospital	No data relevant to review objective. Study addresses neural tube defects including microcephaly. No details were provided for each defect
92	Von Eije 2015	[Imported Zika virus infection in the Netherlands]	Dutch	Journal Article	Hospital	No data relevant to review objective. Study addresses Zika virus and clinical presentation in a non-pregnant 60 year old woman
93	Couture 1983	The use of ultrasound in cerebral malformations	French	Journal Article	Unclear	Prenatal only, focus on diagnosis ofneural tube defects such as dandy Walker syndrome, corpus callosum agenesis
94	Campogrande 1980	The role of echography in prenatal diagnosis	Italian	Journal Article	Unclear	Prenatal comparison only, the study focuses on diagnosis of neural tube defects such as spina bifida, limited information available
95	Pinto Laso 1983	Use of ultrasonography of the skull in the fetus and the newborn infant	Spanish	Journal Article	unclear	Prenatal comparison only, study focuses on neural tube defects such as Dandy Walker syndrome
96	Campbell 1983	Use of ultrasonography of the skull in the fetus and the	USA	Journal Article	Hospital	No information on fetal biometric parameter assessed. Study provides information on prenatal and postnatal correlation, however, the reference

		newborn infant				parameters employed were unclear
97	Snijders 1994	Fetal biometry at 14-40 weeks' gestation	UK	Journal Article	Hospital	No primary data. A fetal assessment chart for normal ranges for fetal biometry generated from 19154 pregnancies
98	Berger 2009	Prenatal Microcephaly: Can We Be More Accurate?	Israel	Journal Article	Hospital	No information on fetal biometric parameter assessed. Case report on four cases of suspected microcephaly
99	Melamed 2011	Sonographic estimation of fetal head circumference: how accurate are we?	Israel	Journal Article	Hospital	No information on fetal biometric parameter assessed. Retrospective cohort study for which prenatal and postnatal measurements were compared. In this study, sonographic ultrasound measurements of head circumference consistently underestimated actual head circumference measured postnatally (mean simple error, -13.6 mm; 95% CI, -13.2 to -13.9) and the difference increased with gestational age
100	Gustavo Malinger 2002	A normal second trimester ultrasound does not exclude intracranial structural pathology	Israel	Journal article	Hospital	No information on fetal biometric parameter assessed. A retrospective study which reported on the prenatal diagnosis of 34 fetuses and findings at 1st trimester, 2nd trimester relative to birth (postnatal or postmortem). Various intracranial structural pathologies were diagnosed following a normal 2nd trimester ultrasound examination and confirmed at birth, just one case of MCP
101	Bromley 1995	Difficulties in the Prenatal Diagnosis of Microcephaly	Italy	Journal Article	Hospital	No information on fetal biometric parameter assessed. Case report of five cases in which sonographic assessments of microcephaly at <22 weeks GA compared to microcephaly confirmation at birth. 1 fetus was diagnosed as MCP and confirmed at

						birth
102	Pennesi 2014	The differential diagnosis of fetal microcephaly			Multicenter hospital	No information on fetal biometric parameter assessed. Cohort study in which 1930 obstetric ultrasound examinations were assessed for diagnostic accuracy of fetal parameters for microcephaly

Abbreviations: FGR (Fetal Growth Restriction), FN (False Negative), FP (False Positive), GA (Gestational Age), MCP (Microcephaly), MRI (Magnetic Resonance Imaging), SGA (Small Gestational Age), US (Ultrasound), TN (True Negative), TP (True Positive).
